# Anti-Platelet factor 4 immunothrombosis—not just heparin and vaccine triggers

**DOI:** 10.1016/j.rpth.2025.102729

**Published:** 2025-03-12

**Authors:** Luisa Müller, Jing Jing Wang, Venkata A.S. Dabbiru, Thomas Thiele, Linda Schönborn

**Affiliations:** 1 Institut für Transfusionsmedizin, Universitätsmedizin Greifswald, Greifswald, Germany; 2 Department of Immunology, College of Medicine and Public Health, Flinders University and SA Pathology, Bedford Park, South Australia, Australia

**Keywords:** heparin-induced thrombocytopenia (HIT), immunothrombosis, platelet-factor 4, thrombotic thrombocytopenia syndrome (TTS), vaccine-induced immune thrombocytopenia and thrombosis (VITT), VITT-like disorders

## Abstract

Derailments at the tightly regulated interface of blood coagulation and innate inflammatory immune responses can lead to pathologic immunothrombosis. A special subset of immunothrombosis is caused by antibodies against platelet-factor 4 (PF4). Anti-PF4 antibodies triggered by heparin treatment in heparin-induced thrombocytopenia (HIT) are known for more than 50 years. Interest in anti-PF4 disorders rekindled when first cases of vaccine-induced immune thrombocytopenia and thrombosis (VITT) occurred during the worldwide COVID-19 vaccination campaign. During this time new diagnostic procedures were established to identify affected patients and to differentiate between different kinds of anti-PF4 antibodies. This review article gives an overview about the current knowledge of HIT and VITT with concepts of the underlying pathogenesis. In addition to heparin and vaccination as known triggers for HIT and VITT, concepts for other clinical cases with anti-PF4 antibodies are described in more detail. Anti-PF4 antibodies in atypical HIT-like syndromes could be triggered by presentation of various polyanions, eg, in settings of orthopedic surgery or bacterial infections. Anti-PF4 antibodies in acute VITT-like disorders can occur after viral infections. Chronic VITT-like anti-PF4 antibodies causing recurrent thrombosis and thrombocytopenia are often linked to monoclonal gammopathies. For all disorders with anti-PF4 antibodies, timely identification in patients with thrombocytopenia with or without thrombosis is crucial for successful therapy.

## Essentials

• Antiplatelet factor (PF) 4 disorders are key models for antibody-mediated immunothrombosis.
• If thrombosis and thrombocytopenia occur together, anti-PF4 disorders should be considered.
• Chronic anti-PF4 disorders could be linked with monoclonal gammopathy of thrombotic significance.
• As anticoagulation alone is not sufficient, additional therapies are needed.

## 1 Pathomechanisms of Immunothrombosis

**Figure 1:**
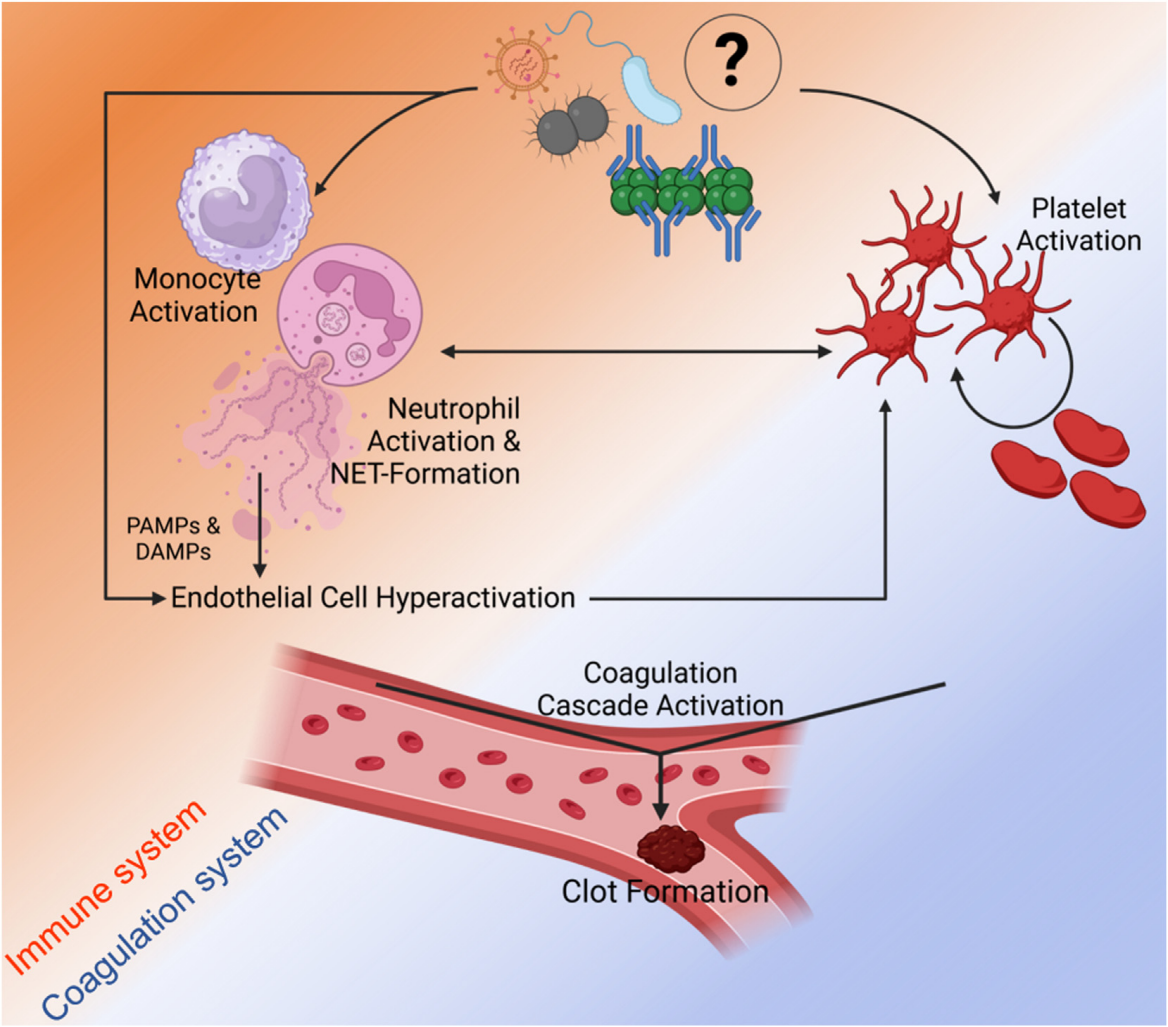
Simplified visualization of immunothrombosis with intertwined mechanisms of the immune (orange) and the coagulation system (blue). The reaction can be triggered by various pathogens, immunocomplexes, or not yet clearly defined other antigens leading to activation of platelets as well as monocytes and neutrophil granulocytes. Briefly, activation of neutrophils subsequently leads to neutrophil extracellular trap (NET) formation, endothelial cell hyperactivation with activation of receptors for pathogen-associated molecular patterns (PAMPs) and/or damage-associated molecular patterns (DAMPs). In combination, this can lead to elevated platelet activation, fuel further NET formation and reinforce coagulation by activation of coagulation factors. Downstream the interplay between immune and coagulation system exaggerates thrombin and fibrin generation leading to clot formation and thrombosis. Moreover, platelets play an important role in immunothrombosis. They have a key role, both in blood coagulation as well as in innate immunity, by using a broad range of receptors on their cell surface to assess and communicate with the surrounding microenvironment. As a reaction to PAMPs, eg, bacterial cell surfaces, platelet binding to neutrophil granulocytes via toll-like receptor 4 is induced [2]. This in turn leads to platelet-enhanced neutrophil activation, eg, via P-selectin glycoprotein ligand 1 [3], and subsequent formation of NETs [4]. NET formation serves as a strong procoagulant and prothrombotic stimulus as neutrophils release a variety of procoagulant factors, eg, von Willebrand Factor as well as chromatin including DNA and histones providing a strongly negative charged surface [5]. Neutrophil activation and NET formation are essential for development of immunothrombosis and can lead to further platelet activation, eg, via thrombin generation [6]. Created in BioRender. Müller L. (2025) https://BioRender.com/e72q127 and modified using Microsoft PowerPoint.

A decade ago, an important concept bridging blood coagulation and innate inflammatory immune responses was termed immunothrombosis by Engelmann and Massberg [1]. Physiologically, the interplay between both pathways is crucial for host defense against various pathogens by hindering pathogen spreading through the blood stream. However, derailments in this tightly orchestrated system can lead to pathologic immunothrombosis, a severe disorder characterized by a prothrombotic profile (Figure 1) [[2], [3], [4], [5], [6]]. In this review, we will highlight that anti-PF4 disorders are prototypic examples for severe antibody-mediated immunothrombosis. The initial trigger for the activation of the cascades finally leading to severe immunothrombosis in anti-PF4 disorders is rather unique and well-defined, ie, platelet-activating anti-PF4 IgG antibodies. However, the downstream mechanisms are probably shared with many other forms of immunothrombosis (eg, in septicemia; during reperfusion injury). Better understanding of the underlying mechanisms of anti-PF4 disorders might also help to better understand other forms of immunothrombosis, especially as newly available monoclonal antibodies can be used to dose-titrate initiation of immunothrombosis and anti-PF4 disorders.

As lately reviewed by Schrottmaier and Assinger [7], activated platelets can critically enhance immunothrombosis by an elevated surface expression of P-selectin or by releasing further signaling molecules, eg, platelet-factor 4 (PF4) [7]. PF4 (also known as CXCL-4) is stored in α-granules of platelets and released upon activation. PF4 is physiologically present as a tetramer with a unique surface charge structure including an arginine-rich and lysine-rich positively charged equatorial band [8]. This allows PF4 to bind with high affinity to negatively charged molecules like heparan sulfate or heparin and exert a procoagulative effect by neutralizing them in plasma and on the cell surface, which lead to decreased antithrombin activity in heparin-anticoagulated patients [9]. PF4 also has direct effects on platelets. It induces platelet activation via the c-Mpl–JAK2 pathway [10]. PF4 released from activated platelets is important for paracrine platelet activation in immunothrombosis and thromboinflammation [11] and in autoimmune conditions like systemic sclerosis [12]. In this narrative review, we aimed to focus on immunothrombosis mediated by anti-PF4 antibodies with special focus on the triggers of these pathologic reactions.

## 2 Heparin-induced Thrombocytopenia

The classic anti-PF4–related immunothrombosis is heparin-induced thrombocytopenia (HIT) and can be seen as a model disease for our understanding of these entities. HIT has been studied for more than 60 years [[13], [14], [15]]. The pathologic trigger of HIT is the anticoagulant heparin, a negatively charged polysaccharide. Thereby, unfractionated heparin (UFH) has a higher risk to cause HIT than low molecular weight heparin (LMWH) due to the different length of the polysaccharide chain [16,17]. Moreover, other negatively charged polyanions like pentosan polysulfate [18] have been shown to induce anti-PF4/polyanion antibody-mediated HIT.

**Figure 2:**
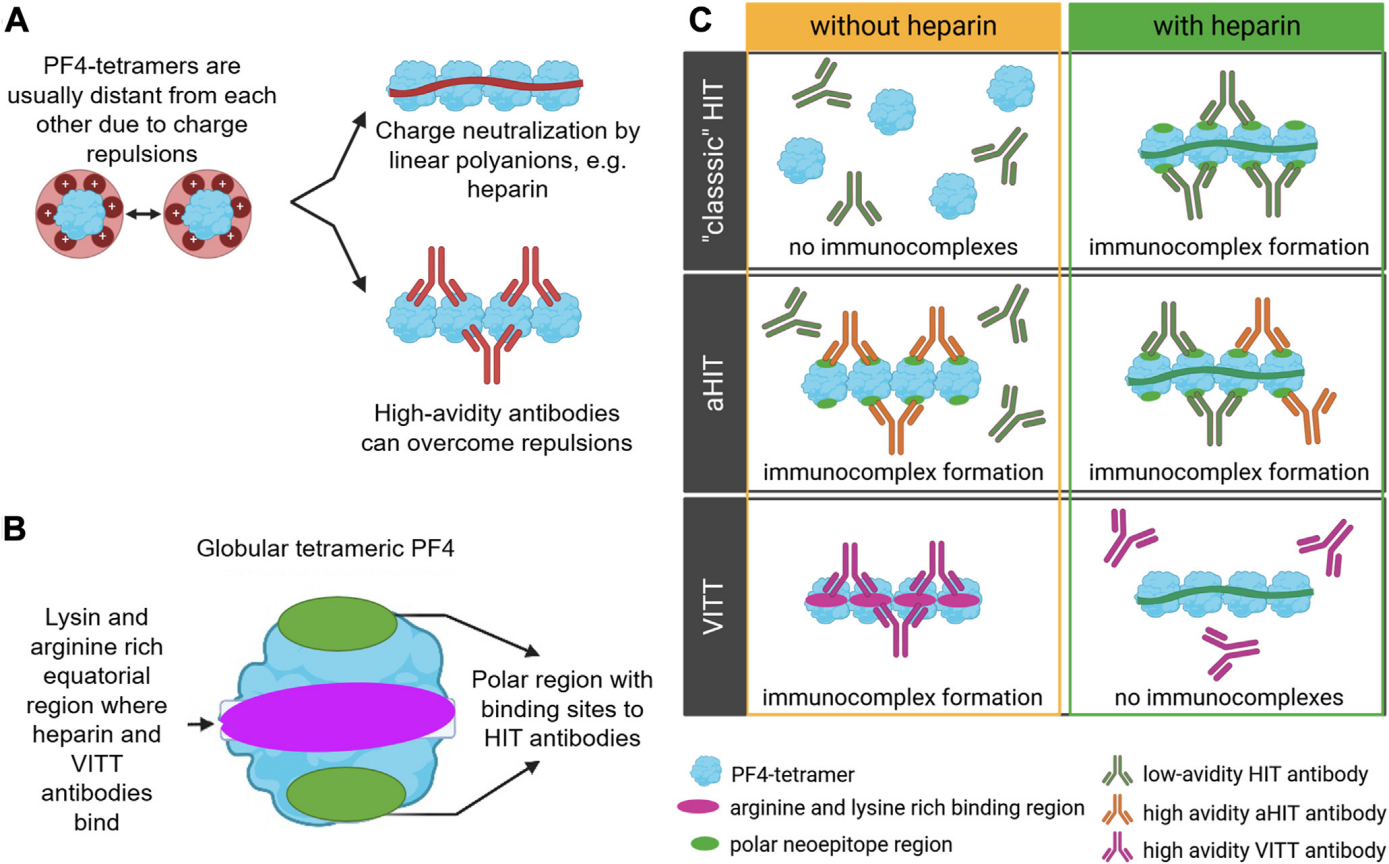
(A) PF4-tetramers are surrounded by a positive charge cloud that leads to repulsions of these molecules. These charge-related repulsions could be overcome either by charge neutralizations by linear polyanions or by high avidity antibodies, which subsequently leads to conformational change of PF4 (not shown in the figure) and to PF4/IgG complex formation. (B) Detailed scheme of PF4 in its tetrameric form with 2 major binding sites: the polar regions able to bind heparin-induced thrombocytopenia (HIT) antibodies (green) and the lysine-rich and arginine-rich equatorial plane (violet) where heparin as well as vaccine-induced immune thrombocytopenia and thrombosis (VITT) antibodies are able to bind. (C) Formation of large immunocomplexes with PF4 and IgG antibodies in conditions with or without heparin in classic HIT, autoimmune heparin-induced thrombocytopenia (aHIT), and VITT. Figure was created in BioRender. Müller, L. (2025) https://BioRender.com/k76u797 and modified using Microsoft PowerPoint.

PF4 molecules organized in tetramers expose a positive charge on their surface in the equatorial region. Consequently, 2 PF4 tetramers repel each other due to their positive charge (Figure 2A). Charge-dependent binding of heparin to PF4 first allows to assemble PF4 tetramers repetitively with the heparin molecules. Binding of PF4 to heparin exposes neoepitopes on PF4 [19], which are recognized by IgG antibodies (Figure 2B). These conformational changes are associated with a change in the energy status, which is only possible with heparin molecules of chain length ≥10 monosaccharides [11,20,21]. As the PF4 charge repulsions are overcome by heparin-mediated charge neutralization, heparin, PF4, and anti-PF4-IgG antibodies form ultralarge immunocomplexes (Figure 2C, upper panel) [22,23]. These complexes then activate platelets via their FcγRIIA receptors, also known as CD32a [24]. Understanding the pathogenesis of HIT has led to the avoidance of UFH in hospitals wherever possible to reduce the risk of HIT and has increased the awareness in pharmaceutical drug development that strongly negatively charged molecules may increase the adverse effect of HIT.

## 3 Autoimmune HIT-syndromes

Patients with pathogenic anti-PF4 antibodies and a severe but sometimes atypical HIT-like clinical picture were identified, whose HIT-symptoms further worsened despite the discontinuation of heparin. These cases can be further subdivided into following subgroups and are summarized under the term autoimmune HIT (aHIT) [25,26]:

### 3.1 Heparin triggered

1. Delayed-onset HIT: This entity is characterized by an onset of clinical symptoms or a worsening of them several days after stopping heparin treatment [27,28].
2. Heparin-flush HIT: This form occurs after exposure to very small amounts of heparin that were sufficient to trigger an immune response but lead in general to antibodies that are able to activate platelets heparin independently [29,30].
3. Persisting HIT: Thrombocytopenia persists beyond 1 week after stopping heparin [31].

In addition, some patients were described who clinically and serologically resembled HIT patients but were not exposed to heparin at all. These subgroup is called spontaneous heparin-induced thrombocytopenia (spHIT), and depending on the terminology, this group is categorized as an autoimmune HIT syndrome [26] (as anti-PF4 antibodies activate platelets independently of heparin) or otherwise can also be considered as alone-standing, nonheparin-triggered subgroup [25].

### 3.2 Nonheparin triggered

4. Spontaneous HIT (spHIT): In contrast to the other subgroups, in this rare disorder, there was no proximate exposure to heparin. In these cases, mostly, complexes of PF4 and polyanions other than heparin (eg, polyphosphates or glycosaminoglycans) are the immunogenic agents that form immunocomplexes with PF4 and anti-PF4/polyanion antibodies. In these patients, other relevant cofactors favoring the formation of pathogenic PF4/polyanion complexes resembling HIT are preceding such as bacterial infections or knee replacement surgery [32,33], possibly leading to thromboinflammation by excessive presentation of pathogen-associated molecular patterns and damage-associated molecular patterns.

In aHIT, antibodies against PF4 are found to be heparin independent and sometimes in coexistence with heparin-dependent anti-PF4 antibodies. In contrast to classic HIT, immune complex formation is facilitated by high avidity antibodies that are able to overcome the charge-related repulsions of PF4 tetramers, leading to conformational changes and the presentation of neoepitopes (Figure 2C, middle panel). These antibodies have such a high avidity that no other cofactors (such as heparin) are required anymore [34].

## 4 Vaccine-induced Immune Thrombocytopenia and Thrombosis

Anti-PF4 antibody-mediated immunothrombosis gained major attention in 2021, when it was identified as cause of a rare but severe adverse reaction to adenoviral vector-based COVID-19 vaccines: vaccine-induced immune thrombocytopenia and thrombosis (VITT) [[35], [36], [37]]. In VITT, previously healthy vaccines developed life-threatening thrombosis at unusual sites (eg, cerebral venous sinus thrombosis [CVST] or splanchnic vein thrombosis) 5 to 30 days after COVID-19 vaccination [38,39]. Anti-PF4 antibodies as early as 4 to 5 days after vaccination indicate that VITT, similar to HIT [40], is a secondary immune response; however, the previous stimulus is not yet identified. Free viral proteins and human cell-line proteins in the vaccine, as well as EDTA (present in the ChAdOx1 nCoV-19 vaccine), contribute to a proinflammatory milieu, which is most likely required for the anti-PF4 immune response [41,42].

The anti-PF4 antibodies in VITT recognize a different epitope on PF4 than that in HIT (Figure 2B). The antibody-binding site of VITT antibodies on PF4 is largely overlapping with the arginine-rich and lysine-rich equatorial region of the heparin-binding site [43,44], which explains the competitive inhibition of VITT antibody binding in the presence of heparin (Figure 2C, lower panel). VITT antibodies typically recognize PF4 alone, whereas HIT antibodies recognize PF4/polyanion complexes (Figure 2). This is important for laboratory testing, as PF4 needs to be added to functional assays [45].

All patients with VITT show the same genetic predisposition: the IGLV3-21∗02 haplotype of the hypervariable region of the IgG light chain [46,47]. An additional specific somatic mutation at position 31 in the IGLV3-21∗02 light chain results in an increase in antibody avidity [47]. Such hypermutations typically occur in B cells during a secondary (boosted) immune response. The coincidence of these separate events likely explains the low incidence of VITT. Whether some HLA haplotypes are overrepresented in VITT is still under discussion [48,49].

Since mRNA vaccines are available as alternatives, adenovirus vector-based COVID-19 vaccines are hardly used anymore in Europe and North America, so VITT has become a real rarity. Nevertheless, adenoviral vector platforms are a promising, affordable platform for vaccine development, especially in low-income and middle-income countries [50]. Deeper understanding of underlying pathomechanisms of VITT thus holds crucial implications for future vaccine development. One of the most important open questions in VITT research to date is the identification of the VITT-triggering antigen(s) contained in the vaccine. This could enable researchers to develop adenovirus vector-based vaccines without a risk for VITT in future.

On the contrary, some VITT cases have been observed after vaccination with non–vector-based COVID-19 vaccines [[51], [52], [53]] and after vaccination against human papillomavirus [54,55]. However, whether these cases are caused by vaccination or whether they reflect aHIT-like pathomechanisms independent from vaccination will need to be further investigated. First published insights from See et al. [56] hint to a frequency of anti-PF4 disorders following non-adenoviral vaccination that align with incidences observed for spHIT and might only reflect “background” spHIT rather than real VITT [56].

## 5 Acute VITT-like Disorders (Postviral VITT)

### 5.1 Terminology and molecular background

Based on the knowledge obtained during the last years of VITT research and an increased awareness for anti-PF4 antibody–associated immunothrombosis, patients were recognized, who presented after natural adenovirus infections with a clinical and serologic VITT-like picture, but without previous exposure to vaccines [[57], [58], [59], [60], [61]]. These newly discovered disorders were initially referred to as VITT-like disorders due to their clinical and serologic similarity to VITT after vaccination.

Interestingly, recent discoveries have shown that the anti-PF4 antibodies of VITT after vaccination and after adenoviral infections are nearly identical down to a common genetic antibody fingerprint [47]. Like the anti-PF4 antibodies of patients with VITT, the antibodies of patients with VITT-like syndrome are monoclonal to oligoclonal [62]. By affinity purification of anti-PF4 antibodies from both patient groups and subsequent sequencing by mass spectrometry, we determined the amino acid sequence of the antigen-binding site of these antibodies. The anti-PF4 antibodies of both patient groups have very similar amino acid sequences at their antigen-binding site to PF4. The antibodies of all examined patients express the IGLV3-21∗02 allele in their light chains and all show a basic lysine (K) to acidic glutamic acid (E) mutation at position 31 (K31E) in the light chain in addition to the ED amino acid motif in their heavy chains. These specific changes result in a sequence of consecutive negatively charged amino acids, which form a negatively charged patch in which the positively charged PF4 tetramer binds. Although these similarities point toward a shared pathomechanism, it is still not clear which antigen(s) in the vaccine or the intact adenovirus triggers the anti-PF4 response.

Assuming that VITT and VITT-like syndromes can be triggered either by virus or vector-based vaccines and show identical molecular characteristics [47], it seems appropriate to broaden the term VITT to vaccine-induced and virus-induced immune thrombocytopenia and thrombosis. However, if explicitly only 1 of the 2 subgroups should be addressed in further studies, we suggest to use the terms postviral VITT and postvaccine VITT [63].

**Figure 3:**
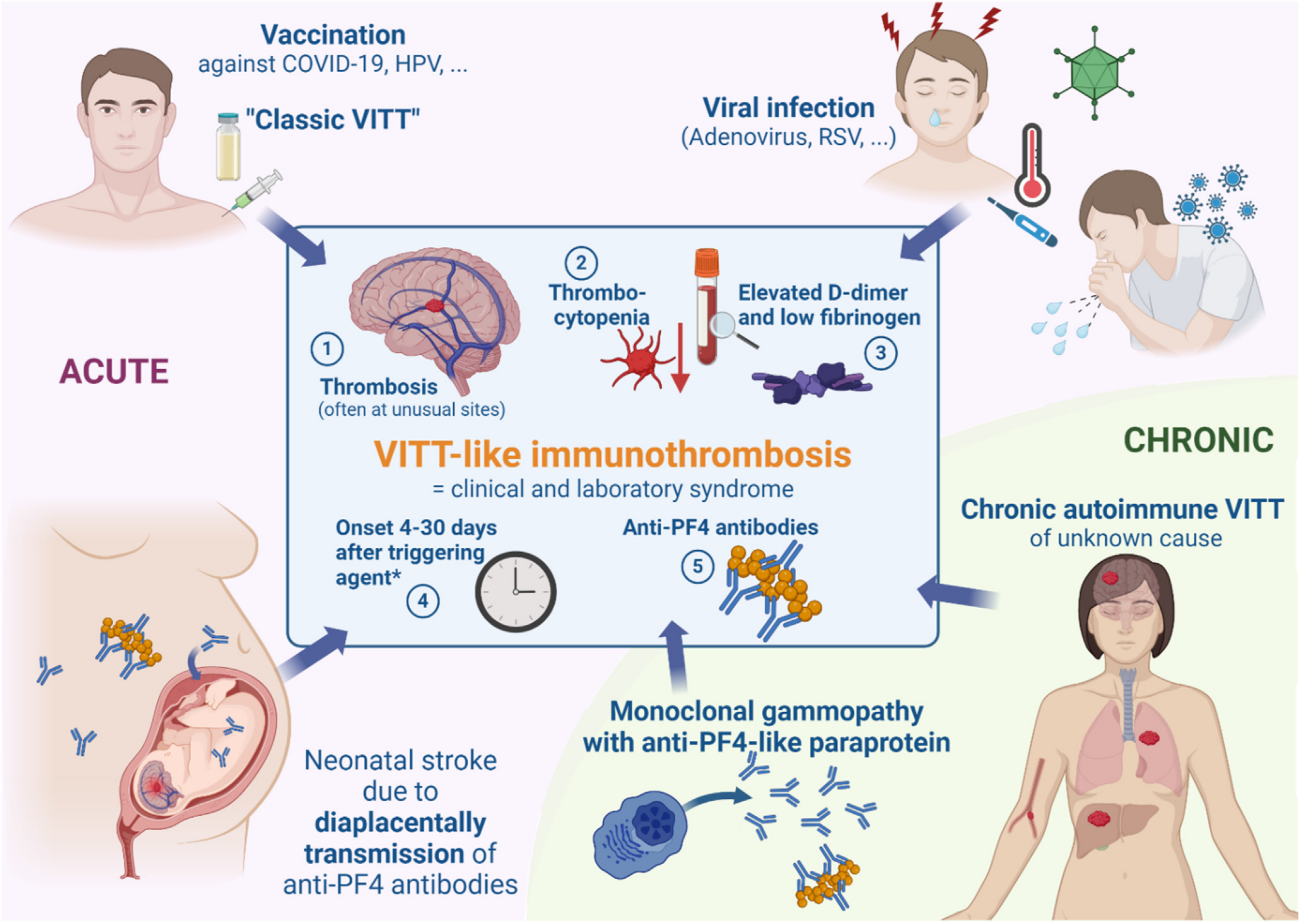
Overview on the clinical presentation and possible triggers of classic VITT and VITT-like syndromes. VITT-like immunothrombosis is a clinically and serologically defined syndrome with 5 typical characteristics: (1) thrombosis, often at unusual sites as cerebral venous sinus thrombosis or portal vein thrombosis; (2) thrombocytopenia; however, in individuals with a very high platelet count only a >50% drop in platelet count might be observed and criteria for thrombocytopenia are not met; (3) typically, VITT-like patients present with highly elevated D-dimer and low fibrinogen levels; (4) the onset of clinical symptoms and/or thrombocytopenia is typically between 4 and 30 days after exposure to the triggering factor (∗only applicable for acute VITT-like syndromes, not chronic); and (5) detection of platelet-activating anti-PF4 antibodies. For screening anti-PF4/heparin or anti-PF4 antigen assays can be used [[64], [65], [66]]. Please note, that some of the rapid assays commonly used in HIT diagnostics do not detect VITT antibodies [45]. For confirmation of the diagnosis “VITT-like syndrome,” a functional assay needs to be performed (eg, PF4-induced platelet activation assay or the PF4-enhanced SRA) [45], especially to exclude crossreactive antibodies with heparin. After adenovirus vector-based COVID-19 vaccination had been identified as triggering agent for classic VITT in 2021, other causes for VITT-like syndromes have been recognized. Viral infections can induce platelet-activating anti–PF4 antibodies with consecutive life-threatening thrombosis in patients of all age groups [[59], [60], [61],[67], [68], [69]]. Anti–PF4 antibodies were shown to be transmitted via the placenta and cause a VITT-like syndrome with stroke in a newborn [70]. In addition, patients with persisting platelet-activating anti–PF4 antibodies were observed, which cause recurrent thromboses and thrombocytopenia. In a subset of them, the paraprotein of their monoclonal gammopathy was identified to have anti-PF4 antibody-like features. HPV, human papilloma virus; PF4, platelet factor 4; RSV, respiratory syncytial virus; VITT, vaccine-/virus-induced immune thrombocytopenia and thrombosis. Created in BioRender. Schönborn, L. (2025) https://BioRender.com/s92v743.

Vaccination-independent VITT-like disorders can be subdivided into 2 main groups: acute VITT-like disorders (mostly postviral VITT) and chronic VITT-like disorders with recurrent episodes of thrombosis and thrombocytopenia (Figure 3) [[64], [65], [66], [67], [68], [69], [70]].

### 5.2 Clinical presentation of acute VITT-like disorders

VITT after COVID-19 vaccination raised awareness for the co-occurrence of thrombosis and thrombocytopenia as an indication of an anti-PF4 disorder. Since then, several patients have been identified with an acute, typical VITT-like pattern, both clinically and serologically. An overview of published cases of patients with acute VITT-like syndrome with different etiology is shown in Table 1 [[57], [58], [59], [60], [61],[67], [68], [69], [70], [71], [72]], being described in more detail in the following paragraphs.

**Table 1:** Published patients with acute VITT-like disorders.

Age (y)	Sex	Clinical features	Possible immunogenic agent	Therapy	Outcome	Reference
5	M	CVST; thrombocytopenia (10 × 10^9^/L); hypofibrinogenemia (83 mg/dL); elevated D-dimer (84.2 mg/L)	Human adenovirus	Multiple platelet transfusions, single 20-g dose IVIG, prednisone, intravenous UFH, bivalirudin	Fatal	[67]
58	F	Multiple arterial strokes, myocardial infarction, and multiple DVT; thrombocytopenia (10 × 10^9^/L); hypofibrinogenemia (98 mg/dL)	Human adenovirus	IVIG on 2 consecutive days, replacement fluid for therapeutic plasma exchange 5% albumin, dexamethasone, prednisone, argatroban	Recovery	[67]
40	F	Intracranial bleeding, PE, portal and popliteal vein thromboses	Human adenovirus	Therapeutic plasma exchange, high-dose IVIG, glucocorticoids, fondaparinux, later rivaroxaban	Recovery	[57]
31	F	DVT, CVST with secondary bleeding; thrombocytopenia (15 × 10^9^/L); elevated D-dimer (>35 mg/L); decreased fibrinogen (60 mg/dL)	Preceding upper respiratory tract infection	Fibrinogen and LMWH, after spontaneous HIT diagnosis change to danaparoid	Fatal	[59,71]
26	M	CVST with secondary bleeding; thrombocytopenia (38 × 10^9^/L); elevated D-dimer (>20 mg/L); decreased fibrinogen (80 mg/dL)	Preceding flu-like symptoms (2 wk)	UFH, after spontaneous HIT diagnosis change to fondaparinux, prednisolone	Recovery; persistent sequelae from CVST	[58,59,68]
70-80^a^	F	PE; thrombocytopenia (22 × 10^9^/L); elevated D-dimer (>20 mg/L)	Not defined	UFH, fondaparinux, apixaban	Recovery	[59]
60-70^a^	M	Stroke with secondary intracranial bleeding; thrombocytopenia (41 × 10^9^/L)	Not defined	Plasma exchange, fondaparinux, apixaban	Recovery; persistent sequelae from stroke	[59]
20-30^a^	F	CVST with secondary bleeding, PE, PVT; thrombocytopenia (43 × 10^9^/L); elevated D-dimer (>30 mg/L)	Human respiratory syncytial virus	UFH	Recovery	[59]
60-70^a^	M	DVT, PE; thrombocytopenia (55 × 10^9^/L); elevated D-dimer (124 mg/L)	Not defined, possibly related to preceding urinary tract infection	Patient refused further therapy	Fatal	[59]
5-10^a^	M	CVST; thrombocytopenia (49 × 10^9^/L); elevated D-dimer (>35 mg/L)	Human adenovirus	LMWH, rivaroxaban, dexamethasone, acetazolamide	Recovery	[59]
7	F	Intracranial hemorrhage, CVST in superior sagittal sinus, retroperitoneal bleeding and subsequent hemorrhagic shock; thrombocytopenia (11 × 10^9^/L); elevated D-dimer (54 mg/L); decreased fibrinogen (40 mg/dL)	Human adenovirus	Thrombectomy, platelet and erythrocyte transfusions, UFH, high-dose IVIG (45 g) on 2 consecutive days, LMWH	Recovery; mild word-finding difficulties; rest tremor but normal muscle tone and strength; concentration and perseverance still reduced	[60]
57	F	Small, atypical left-sided ICH, CSVT, bilateral peripheral and central PE; thrombocytopenia (15 × 10^9^/L); elevated D-dimer (35 mg/L)	Not defined	Thromboprophylaxis with LMWH switched to therapeutic-dose argatroban	Recovery; clinical improvement with mild motoric aphasia and slight hemiparesis; normal platelet counts; vanished anti-PF4 antibodies after 4 mo	[69]
8	F	CVST; thrombocytopenia (50 × 10^9^/L); elevated D-dimer (>36 mg/L)	Adenovirus	LMWH, IVIG	Recovery	[61]
4	F	CVST; thrombocytopenia (23 × 10^9^/L); elevated D-dimer (>35 mg/L)	Rhinovirus-enterovirus-adenovirus	LMWH, IVIG, steroids, platelet transfusion	Recovery	[61]
5	F	CVST; thrombocytopenia (32 × 10^9^/L); elevated D-dimer (28 mg/L)	No virus detectable, streptococcal pharyngotonsillitis	LMWH, later fondaparinux; IVIG, steroids, platelet transfusion	Recovery	[61]
0	M	Neonatal stroke (CVST and arteria stroke) with secondary intracranial bleeding; thrombocytopenia (32 × 10^9^/L); elevated D-dimer	Diaplacentally transmitted anti-PF4 antibodies of a mother with chronic autoimmune anti-PF4 disorder due to paraproteinemia	Phenobarbital due to seizures; platelet transfusion; neither anticoagulation nor steroids or IVIG	Recovery	[70]
2	F	CVST, catheter-associated DVT, intracardiac thrombosis; thrombocytopenia (18 × 10^9^/L); elevated D-dimer (>20 mg/L); decreased fibrinogen (134 mg/dL)	Adenovirus	IVIG, transfusion of platelet concentrates and fresh frozen plasma, TPE, and corticosteroids; switch from heparin to bivalirudin	Recovery, remained thrombosis free on warfarin for 8 y	[72]
5	M	Bilateral lower extremity and subclavian DVT, PE, thrombosis of inferior vena cava and right atrium; thrombocytopenia (87 × 10^9^/L); elevated D-dimer (>5 mg/L)	Parainfluenza	Initially treated with enoxaparin, TPA thrombolysis; later switch to bivalirudin, corticosteroids, TPE	Recovery, remained thrombosis free on warfarin for 8 y	[72]
4	F	Femoral DVT, upper extremity DVT, iliac thrombosis, thrombosis of inferior vena cava and right atrium; thrombocytopenia (38 × 10^9^/L); elevated D-dimer (>20 mg/L); decreased fibrinogen (144 mg/dL)	Not defined (2 wk after otitis media and febrile upper respiratory illness)	Heparin and TPA thrombolysis, switch to bivalirudin; corticosteroids, TPE, rituximab	Recovery, remained thrombosis free on warfarin for 8 y	[72]

CVST, cerebral venous sinus thrombosis; DVT, deep vein thrombosis; F, female; ICH, intracerebral hemorrhage; IVIG, intravenous immune globulin; LMWH, low molecular weight heparin; M, male; PE, pulmonary embolism; PVT, portal vein thrombosis; TPA, tissue-type plasminogen activator; TPE, therapeutic plasma exchange; UFH, unfractionated heparin; VITT, vaccine-induced immune thrombocytopenia and thrombosis.

a For data protection reasons, only a narrow age range was given in the original article.

#### 5.1.1 Postviral VITT

Adenovirus infection: The diagnosis of postviral VITT has been made also in retrospect in patients with severe thrombotic complications long before the SARS-CoV-2 pandemic, taking advantage of repository samples [59]. Most likely, also some of the previously described cases of aHIT and spHIT might have been VITT-like disorders.

Adenoviral infections were recognized as the most likely cause of these acute anti-PF4 antibodies. One of the first published cases by Warkentin et al. [67] shows the typical clinical and serologic characteristics of virus-associated VITT-like disorders, which are summarized in Figure 3. The authors described a 5-year-old boy who presented with thrombocytopenia 5 days after the viral symptoms of an adenoviral gastrointestinal infection had started. The typical time window, which is already known from HIT [73] and VITT [74], of at least 4 to 5 days between the triggering agent and onset of thrombocytopenia or thrombosis is also present in VITT-like disorders and reflects the time that is needed to produce relevant titers of anti-PF4 antibodies. Three days later, the patient developed severe headache, the most commonly described symptom in patients with acute postvaccine and postviral VITT [38,59,60,75]. Cranial imaging revealed CVST. In addition to thrombocytopenia, which was refractory to repeated platelet transfusions, the laboratory parameters also included markedly elevated D-dimer and low fibrinogen. Subsequently, typical VITT-like antibodies were detected in this patient by a positive fluid-phase ELISA [58] and a positive PF4-enhanced serotonin release assay (SRA). Despite anticoagulation with UFH, which was later switched to bivalirudin, and intravenous immunoglobulin (IVIG) on day 11, this patient unfortunately died due to the secondary complications of CVST 13 days after his viral symptoms had started. Reviewing other case series of children with CVST caused by VITT-like anti-PF4 antibodies [[59], [60], [61]] show a common pattern: rapid recognition of such patients and immediate initiation of therapy with therapeutic-dose anticoagulation and interference with the mechanism of platelet activation, eg, by high-dose IVIG, prevent progression of thrombotic complications.

This, however, creates a clinical dilemma. Especially, the symptoms of adenovirus infections are the common cold. Headache is frequent in patients with common cold and usually harmless. Adult patients described that the headaches associated with VITT and VITT-like disease are extremely severe, probably the most severe headaches they ever experienced in life [75]. Children will not be able to give this information. Starting therapeutic-dose anticoagulation in any child presenting with somewhat more severe symptoms of the common cold would result in overtreatment. One solution would be to test patients with a high clinical suspicion of a VITT-like disorder for anti-PF4 antibodies in an appropriate screening assay [59,76]. If anti-PF4 antibody screening assays are not available, testing for D-dimer could be an alternative. However, D-dimer are much less specific, and weakly to moderately elevated D-dimer do not indicate VITT. D-dimer are expected to be highly elevated in a patient with a VITT-like disorder [59,67].

Importantly, VITT-like anti-PF4 antibodies are not recognized by some commercially available rapid assays, especially chemiluminescence assays [59,76], and show negative or only weakly positive results in functional assays in the presence of heparin (eg, heparin-induced platelet activation [HIPA] assay or SRA). A novel rapid chemiluminescence assay that is able to distinguish between anti-PF4/heparin and anti-PF4 antibodies may allow a rapid therapeutic decision [59]. For confirmation of diagnosis, special platelet activation assays are necessary, eg, the PF4-induced platelet activation (PIPA) assay [35] or the PF4-enhanced SRA [77,78].

As we already know from HIT [73] and VITT after COVID-19 vaccination [79], platelet-activating VITT-like antibodies are transient in the majority of patients. They lose their ability to activate platelets usually after some months. The clinical and serologic similarity between VITT after vaccination and VITT-like syndromes after adenoviral infection suggests a common origin of these 2 misdirected autoimmune disorders. It is reasonable to suspect that it is a component of the adenovirus, as this is present in both situations. Interestingly, adenoviruses are capable to activate platelets themselves, supposedly via Coxsackie and adenovirus receptor, and activate endothelium of the cerebral vein sinus via Coxsackie and adenovirus receptor [80]. This might be the link to the observed CVST that is a rather unusual site for classic thrombosis, but typically seen in many patients with VITT-like disorders.

#### 5.1.2 Other triggers for VITT

VITT-like disorders have also been observed after HPV vaccination [54,55] or in a patient after infection with respiratory syncytial virus [59]. Thus, VITT-like syndromes do not seem to be limited to contact with adenoviruses.

#### 5.1.3 Neonatal VITT-like disease

The recently published case by Häusler et al. [70] has shown that an acute VITT-like syndrome can also be caused by passive transfer of anti-PF4 antibodies. The authors reported on a baby with neonatal CVST, arterial stroke, and secondary intracranial bleeding in whom diagnostic workup revealed platelet-activating anti-PF4 antibodies that were diaplacentally transferred from the 35-year-old mother. These antibodies were apparently chronically present in the mother as she had a history of thrombosis.

### 5.3 Treatment options for VITT-like disorders

The treatment of VITT-like syndromes requires a multifaceted approach and is based on all the experience gained in patients with classic VITT following vaccination.

#### 5.3.1 Anticoagulation

Anticoagulation is one of the important corner stones [81,82]. Non-heparin anticoagulants are preferred, as crossreactivity with PF4/heparin complexes cannot easily be assessed or ruled out by using a screening assay. To ensure that the patient’s antibodies have no platelet-activating crossreactivity with PF4/heparin complexes, a functional assay must be performed.

Parenteral direct thrombin inhibitors argatroban and bivalirudin are preferred in intensive care due to their rapid anticoagulation effects and the ability to quickly adjust dosing, especially in cases of high bleeding risk or need for urgent surgery [82].

Danaparoid not only is successfully used to treat HIT [83] but has also been effective for the treatment of VITT [84]. Danaparoid acts by inhibiting factor Xa and PF4 complexes, preventing platelet activation.

Heparin is usually avoided in treatment of VITT due to concerns of crossreacting antibodies with PF4/heparin complexes. However, Singh et al. [85] could show that heparin even interferes with the binding of anti-PF4 antibodies and PF4 leading to inhibition of thrombus formation in VITT. Thus, heparin might be an option when no alternative anticoagulation is available or when crossreactivity with heparin was ruled out by a functional assay.

Fondaparinux is also effective in patients with stable VITT but should be avoided in urgent surgical situations due to its long half-life [82].

Direct oral anticoagulants (DOACs), such as rivaroxaban, apixaban, edoxaban, and dabigatran, have been used effectively in patients with postvaccine VITT [[86], [87], [88]]. Initial case reports of patients with postviral VITT also indicate that DOACs are a suitable to treat stable patients especially in the outpatient setting [57,59]. However, in patients with severe anti-PF4 thrombosis or those requiring intensive care, DOACs are not recommended [82].

Vitamin K antagonists should be avoided in acute VITT-like syndromes, as they are suspected to increase the risk of thrombotic complications like skin necrosis [89]. However, they may be considered for long-term anticoagulation once platelet counts normalize.

#### 5.3.2 Therapies to support anticoagulation

Modulating FcγRIIA receptor activation with IVIG is particularly important because patients with VITT and VITT-like disorders usually present only when they already have symptoms of often life-threatening thrombosis [38,39]. IVIG rapidly blocks the binding of anti-PF4 antibodies to platelets’ FcγRIIA receptor, reducing platelet activation and improving platelet counts [89,90]. It is typically used in conjunction with anticoagulation therapy, with a recommended dosing regimen of 1 g/kg bodyweight for 2 consecutive days, and potentially a third dose if the patient is refractory.

In severe or refractory cases, therapeutic plasma exchange may be used to reduce circulating anti-PF4 antibodies and other inflammatory markers, offering symptom relief and survival benefits, particularly when other treatments have failed [91].

Platelet transfusions should be avoided in patients with VITT and VITT-like disorders, as they can exacerbate thrombotic complications. They may be considered in life-threatening bleeding situations.

## 6 Chronic VITT-like Anti-Pf4 Antibodies in Patients with Recurrent Thrombosis

### 6.1 Chronic autoimmune VITT-like disorder

In contrast to the transient character of virus-triggered or passively transmitted anti-PF4 disorders, there is an only recently recognized patient group in whom VITT-like antibodies have persisted over years and have triggered chronic recurrent thrombosis with thrombocytopenia (Table 2) [59,63,[92], [93], [94]]. The clinical course of a particularly severely affected patient is outlined in Lindhoff-Last et al. [94]. The 38-year-old patient experienced multiple episodes of venous and arterial thrombotic complications associated with thrombocytopenia over a period of 15 years while receiving various anticoagulation therapies. Typical VITT-like antibodies could be detected in this patient’s consecutive blood samples from over the last 15 years, which were apparently the cause of the recurrent thrombotic complications. As the patient repeatedly developed thromboses despite various dual anticoagulation and platelet aggregation therapies, the Bruton tyrosine kinase (BTK) inhibitor ibrutinib was considered as rescue therapy. Originally used as a drug for the treatment of lymphomas, it inhibits the Fcγ receptor–dependent signaling pathway of platelet activation, in which BTK plays an important role [95,96]. The patient’s clinical picture improved significantly under 280 mg ibrutinib once a day in parallel with therapeutic anticoagulation. The platelet count and D-dimer reached normal values, and the patient’s clinical symptoms improved significantly. The inhibitory effect of ibrutinib on platelet activation could be visualized in immunofluorescence microscopy, where activation markers (eg, tethers) had then decreased significantly compared with blood smears from before the start of therapy. It is currently unclear which drivers caused these chronic anti-PF4 antibodies in this patient.

**Table 2:** Published Patients with chronic VITT-like disorders.

Age	Sex	Clinical features	Possible immunogenic agent	Therapy	Outcome	Reference
79	F	Recurrent DVT and PE over 3 y, stroke despite therapeutic anticoagulation (apixaban); low platelet count (81 × 10^9^/L); elevated D-dimer (10.4 mg/L)	Monoclonal gammopathy (monoclonal paraprotein of IgG-κ type with persisting PF4 reactivity)	Multiple anticoagulants and platelet aggregation inhibitors (eg, LMWH later switched to fondaparinux)	Recovery; although still in the lower normal range, platelet count stabilized	[59,92]
64	M	Recurrent breakthrough thrombosis while on anticoagulation (apixaban/warfarin); medical record of PE, PVT, myocardial infarction, splenic infarction; thrombocytopenia (<150 × 10^9^/L)	MGTS with monoclonal paraprotein of IgG-κ type with persisting anti-PF4/polyanion reactivity	Periodic infusions of IVIG, rituximab, daratumumab, and dexamethasone	Recovery, platelet counts still low	[93]
37	F	15 y with recurrent episodes of venous and arterial thromboembolic complications; multiple microembolic strokes developed after acute COVID-19 and recurrent strokes thereafter; thrombocytopenia (<100 × 10^9^/L); elevated D-dimer	Autoimmune anti-PF4 disorder (no paraprotein detectable)	Treatment with high-dose apixaban, aspirin, fondaparinux, and clopidogrel, repeated high-dose IVIG, therapeutic plasma exchanges, ibrutinib	Stable state under ibrutinib	[94]
60	M	4 y with recurrent episodes of venous and arterial thrombotic complications; bilateral lower limb arterial thromboses, recurrent PE, hepatic vein thrombosis, DVT; thrombocytopenia (21 × 10^9^/L); elevated D-dimer	MGTS with monoclonal paraprotein (0.3 g/dL) of IgG-κ type with persisting VITT-like anti-PF4 activity	Therapeutic-dose anticoagulation, due to recurrent thromboses and thrombocytopenia repeated high-dose IVIG; switch to ibrutinib resulted in normalization of chronically elevated D-dimer levels	Stable state under ibrutinib, no new thrombosis (6-mo follow-up)	[63]
46	M	2 y with recurrent episodes of venous thrombotic complications; bilateral adrenal hemorrhage (due to adrenal vein thrombosis), bilateral DVT, bilateral lower limb superficial vein thrombosis, fatal CVST; thrombocytopenia (10 × 10^9^/L); elevated D-dimer	MGTS with monoclonal paraprotein (<0.1 g/dL) of IgG-λ type with persisting VITT-like anti-PF4 activity	Therapeutic-dose anticoagulation with apixaban	Fatal	[63]
35	M	22 y with recurrent episodes of venous and arterial thrombotic complications; mesenteric and splenic vein thrombosis, DVT, recurrent PE, arterial stroke, left ventricular thrombosis, myocardial infarction and intrastent thrombosis; thrombocytopenia (23 × 10^9^/L); elevated D-dimer	MGTS with monoclonal paraprotein (0.7 g/dL) of IgG-λ type with persisting VITT-like anti-PF4 activity	Therapeutic-dose anticoagulation, multiple courses of steroids, therapeutic plasma exchange, IVIG, rituximab; despite that recurrent thromboses and thrombocytopenia; switch to bortezomib, cyclophosphamide, and daratumumab	Stable state with normalization of platelet count and loss of M-protein, no new thrombosis (12-mo follow-up)	[63]
47	F	5 y with recurrent episodes of venous thrombotic complications; superficial vein thrombosis, recurrent PE, lower limb and upper-limb DVT, CVST, renal vein thrombosis; thrombocytopenia (30 × 10^9^/L); elevated D-dimer	MGTS with monoclonal paraprotein (0.1 g/dL) of IgG-λ type with persisting VITT-like anti-PF4 activity	Therapeutic-dose anticoagulation with rivaroxaban (later switched to dabigatran, vitamin K antagonist, or LMWH due to recurrent thromboses despite anticoagulation); multiple courses of steroids, therapeutic plasma exchange, IVIG, rituximab. Due to recurrent thromboses switch to bortezomib, daratumumab, and dexamethason	Stable state, no new thrombosis (3-mo follow-up)	[63]

CVST, cerebral venous sinus thrombosis; DVT, deep vein thrombosis; F, female; ICH, intracerebral hemorrhage; IVIG, intravenous immune globulin; LMWH, low molecular weight heparin; M, male; MGTS, monoclonal gammopathy of thrombotic significance; PE, pulmonary embolism; PVT, portal vein thrombosis; UFH, unfractionated heparin; VITT, vaccine-induced immune thrombocytopenia and thrombosis.

### 6.2 VITT-like monoclonal gammopathy of thrombotic significance

An underlying monoclonal gammopathy with a paraprotein exposing anti-PF4 antibody-like features has been proven to activate platelets and cause recurrent thromboses in different patients [63,92,93]. According to initial studies, the prevalence of platelet-activating anti-PF4 antibodies might range between 1.5% and 3% in patients with monoclonal gammopathy of unknown significance (MGUS) and a history of thrombosis [97]. In addition to the usual diagnostics of thrombophilic risk factors (such as antiphospholipid syndrome), anti-PF4 antibodies should be excluded in a suitable screening assay in all patients with chronic recurrent thromboses. Recognizing these antibodies is particularly important because these rare cases are often difficult to treat and the patients show breakthrough thrombosis even when on anticoagulation [63]. In addition to anti-PF4 antibody screening, protein electrophoresis and immunofixation should be performed to reveal monoclonal gammopathy. In patients whose chronic anti-PF4 thrombosis is caused by a paraprotein, meaning their MGUS has developed into an monoclonal gammopathy of thrombotic significance (MGTS) [98], it may even be necessary to start antimyeloma therapy [63,99]. Interestingly, the anti-PF4 antibodies found in patients with chronic VITT-like MGTS differ from those observed in patients with postvaccine VITT or postviral VITT. These antibodies do not exhibit the nearly identical fingerprint seen in the anti-PF4 antibodies associated with acute VITT [47]. Instead, the anti-PF4 antibodies in patients with chronic VITT-like MGTS display distinct and individually unique amino acid sequences. While the binding epitopes of these antibodies on PF4 differ from those of anti-PF4 antibodies in patients with acute postvaccine or postviral VITT, they share similar negatively charged antigen-binding sites (paratopes) [63].

## 7 Conclusion

HIT-like and VITT-like cases with corresponding anti-PF4 antibodies might be the cause of misdirected immune reactions against bacteria (HIT-like) or viral (VITT-like) infections. Especially during the last 4 years, new diagnostic procedures were established to identify affected patients and even to differentiate between anti-PF4/polyanion and anti-PF4 antibodies. VITT-like disorders require therapeutic options beyond anticoagulation, such as IVIG or therapeutic plasma exchange. In patients with chronic recurrent anti-PF4 thromboses, BTK inhibitors may be beneficial, and if caused by a paraprotein, antimyeloma therapy should be considered. The timely identification of an underlying anti-PF4 disorder in patients with thrombocytopenia with or without thrombosis is crucial for successful therapy.
